# Intestinal parasites and vector-borne pathogens in stray and free-roaming cats living in continental and insular Greece

**DOI:** 10.1371/journal.pntd.0005335

**Published:** 2017-01-31

**Authors:** Anastasia Diakou, Angela Di Cesare, Paolo Matteo Accettura, Luciano Barros, Raffaella Iorio, Barbara Paoletti, Antonio Frangipane di Regalbono, Lénaïg Halos, Frederic Beugnet, Donato Traversa

**Affiliations:** 1 Laboratory of Parasitology and Parasitic Diseases, School of Veterinary Medicine, Faculty of Health Sciences, Aristotle University of Thessaloniki, Thessaloniki, Greece; 2 Faculty of Veterinary Medicine, University of Teramo, Località Piano D’Accio snc., Teramo, Italy; 3 Faculdade de Medicina Veterinaria MSV/UFF, Rua Vital Brasil Filho, 64 Icaraí, Niterói, Rio de Janeiro, Brazil; 4 Department of Animal Medicine, Production and Health, University of Padua, Viale dell’Università 16, Legnaro, Padua, Italy; 5 Merial S.A.S, 29 Av Tony Garnier, Lyon, France; Fondation Raoul Follereau, FRANCE

## Abstract

This survey investigated the distribution of various intestinal parasites and vector-borne pathogens in stray and free-roaming cats living in four regions of Greece. A total number of one hundred and fifty cats living in three Islands (Crete, Mykonos and Skopelos) and in Athens municipality was established as a realistic aim to be accomplished in the study areas. All cats were examined with different microscopic, serological and molecular assays aiming at evaluating the occurrence of intestinal parasites, and exposure to or presence of vector-borne infections. A total of 135 cats (90%) was positive for one or more parasites and/or pathogens transmitted by ectoparasites. Forty-four (29.3%) cats were positive for one single infection, while 91 (60.7%) for more than one pathogen. A high number of (n. 53) multiple infections caused by feline intestinal and vector-borne agents including at least one zoonotic pathogen was detected. Among them, the most frequently recorded helminths were roundworms (*Toxocara cati*, 24%) and *Dipylidium caninum* (2%), while a high number of examined animals (58.8%) had seroreaction for *Bartonella* spp., followed by *Rickettsia* spp. (43.2%) and *Leishmania infantum* (6.1%). DNA-based assays revealed the zoonotic arthropod-borne organisms *Bartonella henselae*, *Bartonella clarridgeiae*, *Rickettsia* spp., and *L*. *infantum*. These results show that free-ranging cats living in areas of Greece under examination may be exposed to a plethora of internal parasites and vector-borne pathogens, some of them potentially able to infect humans. Therefore, epidemiological vigilance and appropriate control measures are crucial for the prevention and control of these infections and to minimize the risk of infection for people.

## Author Summary

Several intestinal parasites and vector-borne pathogens, some of that with zoonotic potential, often occur in free-ranging cat populations. Stray cats are abundant in touristic areas of Greece, where they live in free colonies. With the aim to enhance our knowledge on the hazard posed by cats living in Greek touristic spots, the present research has evaluated the presence of intestinal parasites and vector-borne pathogens in stray cats living in selected areas. The study demonstrates that these pathogens are present in Greece and that appropriate control measures should be implemented to minimize the risk for cats and people.

## Introduction

The domestic cat (*Felis silvestris catus*) may harbor intestinal parasites and arthropod-borne pathogens (e.g. bacteria, protozoa, and helminths) of high relevance in veterinary and human medicine. Some of these infections may be deadly in cats and are a potential threat for human beings [1].

The past decade has seen a new interest on zoonotic parasites and vector-borne diseases (VBDs) of companion animals [2–4]. Nonetheless, knowledge of various aspects of these infections in feline hosts requires improvements [3], especially in terms of occurrence and distribution in different geographic areas.

In most cases, zoonoses in privately owned cats can be prevented and managed through standard veterinary care, appropriate dewormers and effective ectoparasiticides [1,4], while free-roaming, stray or feral cats receive scarce or no veterinary care. Thus, these may represent a potential health threat to companion animals and people. This is particularly important when people are exposed to potentially contaminated environments and/or come in contact with infected free-roaming cats and vectors. When infected, free-ranging cats have the potential to contaminate the environment with parasitic elements, and could be a source of zoonotic pathogens for vectors, other animals and humans. As key examples, the cat roundworm *Toxocara cati* is an underestimated agent of human diseases [2,4], cat flea-borne bartonellosis and rickettsiosis are (re)emerging threats for people [5–7] and the potential role of cats as infection source for phlebotomine sand flies with *Leishmania infantum* should be taken into account [8].

Stray cats are very abundant and popular in Greece, including touristic areas, where they often live in free colonies managed on a volunteer basis. The presence of intestinal helminths and ectoparasites of zoonotic importance in feline populations of Greece is known [9–11], but a constant update of knowledge is necessary, especially for VBDs. In fact, pathogens transmitted by ectoparasites have recently spurred attention, especially for a potential geographic dissemination into both endemic and formerly unaffected regions. This is of importance considering that VBDs of cats are transmissible to humans *via* the bite of ectoparasites (e.g. fleas) and that some (e.g. bartonellosis) may be directly transmitted from an infected cat to a human. Epidemiological attention is thus required in those areas where stray or free-roaming animals are abundant and come in contact with people or owned pets.

The present study evaluated the occurrence of zoonotic parasites and vector-borne pathogens in stray cats living in selected areas of Greece.

## Methods

### Study areas and sampling

**Fig 1:**
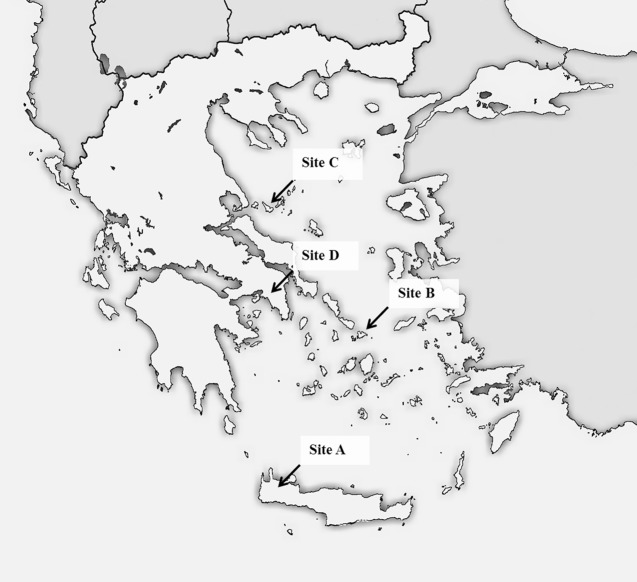
Study areas. Study sites of a multicentric study carried out in Greece to investigate the occurrence of zoonotic intestinal parasites and vector-borne pathogens in cats living in Crete (Site A), Mykonos (Site B), Skopelos (Site C) and Athens (Site D).

In summer 2015, a multicentric study was carried out in four areas of Greece, i.e. the islands of Crete (Site A), Mykonos (Site B) and Skopelos (Site C), and the city of Athens (Site D) (Fig 1).

These sites were selected based on their touristic attractiveness, presence of colonies of stray cats, and willingness of local veterinarians and animal welfare organization to collaborate to the study. Official authorizations to examine and sample the animals were obtained from the local Municipality authorities. Fecal and blood samples were collected from cats either free-roaming or living in colonies from the four aforementioned sites. Faeces were collected using a pediatric clyster, while blood was taken following sedation, whenever necessary.

Available data on gender, breed, living conditions, age, clinical status (presence or absence of clinical signs compatible with parasitoses) were registered for each animal.

Overall, 34, 43, 25, 48 (i.e. total of 150 cats) faecal samples were collected in Site A-D, respectively. Blood samples were also collected from all these animals with the exception of two cats from Site B.

### Faecal samples examination

For each faecal sample a classical floatation technique has been performed as follows. About 5 grams of faeces were diluted in ~20 ml of ZnSO_4_ (33.2% w/v) floatation solution and then the mixture has been strained through a sieve with a mesh width of ~250 μm. The solution was divided into two 15-ml Falcon centrifuge tubes and centrifuged at 2000 rpm for 5 min. A Pasteur pipette was used to transfer a supernatant aliquot from the first tube to a glass slide, which has been examined under a light microscope at 200X and 400X magnifications. Parasitic elements were identified according to morphological and morphometric key features, i.e. egg morphology, wall surface, measures [12]. An aliquot of ~500 μL was transferred from the second Falcon tube to an Eppendorf tube and stored at -20°C pending further analysis. All segments of cestodes found at the macroscopic handling of the faeces were washed in saline and preserved in ethanol 70% for further analysis.

Faecal samples were also been subjected to a copromicroscopic analysis with the Baermann’s technique for investigating the occurrence of pulmonary metastrongyloids. Methodologies and results were previously published [13] and not further discussed here.

### Blood examination

#### Microscopy

Giemsa stained blood smears were also performed as follows. A drop of EDTA-blood was placed on a microscope slide and smeared. After air dry, the glass slides were covered with absolute methanol for 5 min and then air-dried. The glass slides were covered with Giemsa (1:20 in buffered water) and stained for 30 min, washed briefly with distilled water, dried at room temperature and then analyzed by microscopy (1000X magnification).

#### Serology

Sera obtained from blood samples centrifuged at 3000 rpm for 10 min were subjected to the following serological examinations:

- The feline Heartworm Antibody test Kit “*SoloStep FH Cassette*” (HESKA) was used for the qualitative detection of antibodies specific for *Dirofilaria immitis* according to the manufacturers’ instructions.
- Anti-*Bartonella henselae*-IgG-antibodies were detected with the indirect immunofluorescence antibody assay (IFAT) “Mega FLUO BARTONELLA henselae” (Megacor Diagnostik GmbH) kit (cut-off 1:64) according to the manufacturers’ instructions.
- The presence of antibodies against *Rickettsia typhi* and *Rickettsia felis* was evaluated by IFAT using a commercial antigen (slides coated with purified individual substrate antigens of *R*. *typhi* and *R*. *felis—*Fuller Laboratories) and anti-cat IgG (FLUO FITC anti-cat IgG conjugate). According to the literature, a screening dilution of 1:64 was used as cut-off [14].
- Antibodies against *Ehrlichia canis* were investigated by IFAT using a commercial antigen (i.e. slides coated with *E*. *canis* antigens—MegaScreen FLUO *Ehrlichia canis*) and anti-cat IgG (FLUO FITC anti-cat IgG conjugate–Fuller Laboratories). A screening dilution of 1:40 was used as cut-off [15].
- An IFAT test has been performed to evaluate the presence of antibodies against *L*. *infantum* using commercial slides coated with promastigotes (MegaFLUO Leish—Megacor Diagnostik GmbH) and anti-cat IgG (FLUO FITC anti-cat IgG conjugate). A screening dilution of 1:80 was selected as a cut-off [16].

For all IFAT determinations, cat sera were diluted in PBS according to the cut-off and incubated on the individual slides wells for 30 min at 37°C into a humid chamber to allow the antigen-antibody formation. Then, the slides were washed using PBS to remove non-reactive serum proteins, and a fluorescent conjugate has been added to label the antigen-antibody complex. After incubation at 37°C for 30 min into a humid chamber, the slides were washed again to remove the non-reactive conjugate and the resulting reactions were visualized using a standard fluorescent microscopy at 400X magnification. Though some of the aforementioned procedures were used *off label*, all of them have already been formerly used in the serological diagnosis of cat infections [14–16].

#### Molecular examination

All blood samples were subjected to different molecular examinations, in order to identify, if present, the pathogen/s at the species level.

Genomic DNA was extracted using a commercial kit (QIAamp DNA blood Mini kit—Qiagen GmbH, Hilden, Germany) from all blood samples according to the manufacturer’s instructions.

Positivity to *Bartonella* spp. [17], Anaplasmataceae [17], *Rickettsia* spp. [17,18], and *Leishmania* spp. [17,18] DNA was tested by different PCR protocols available in the literature.

PCR products were visualized under UV illumination after electrophoresis migration on a 1.8% gel agarose, stained with GelRed (Promega), and then individually sequenced directly in an automated sequencer. Sequences were determined in both directions, aligned using ClustalX software and compared with those available in GenBank using Nucleotid Basic Local Alignment Search Tool (BLASTN) [19].

## Results

### Faeces

Overall, 150 stool samples were subjected to microscopic examinations, i.e. 34, 43, 25, 48 in Sites A-D respectively.

#### Copromicroscopy (Table 1)

The most common intestinal zoonotic helminth found at faecal examinations was *T*. *cati* (24%), followed by *Dipylidium caninum* (2%). Other common parasites detected were nematodes (e.g. *Toxascaris leonina*– 8%), protozoa (i.e. *Cystoisospora rivolta*– 7.3%).

**Table 1:** 

Pathogen	Site A (n = 34) n/%	Site B (n = 43) n/%	Site C (n = 25) n/%	Site D (n = 48) n/%	Total (n = 150) n/%
*Toxocara cati*	3 (8.8)	14 (32.6)	8 (32)	11(23)	36 (24)
*Toxascaris leonina*	0 (0)	2 (4.7)	0 (0)	10 (20.8)	12 (8)
*Cystoisospora rivolta*	0 (0)	6 (14)	4 (16)	1 (2.1)	11 (7.3)
*Aelurostrongylus abstrusus*	1 (2.9)	3 (7)	2 (8)	5 (10.4)	11 (7.3)
*Cystoisospora felis*	1 (2.9)	4 (9.3)	2 (8)	3 (6.3)	10 (6.7)
*Troglostrongylus brevior*	0 (0)	5 (11.6)	2 (8)	2 (4.2)	9 (6)
*Ancylostoma tubaeforme*	0 (0)	0 (0)	6 (24)	0 (0)	6 (4)
*Uncinaria stenocephala*	0 (0)	0 (0)	2 (8)	0 (0)	2 (1.3)
*Dipylidium caninum*	0 (0)	3 (7)	0 (0)	0 (0)	3 (2)
Other cestodes*	0 (0)	5 (11.6)	5 (20)	5 (10.4)	15 (10)
*Giardia* spp.	0 (0)	0 (0)	0 (0)	1 (2.1)	1 (0.7)
Total number of dogs infected by intestinal/respiratory parasites	4 (11.8)	25 (58.1)	16 (64)	25 (52.1)	70 (46.7)

Microscopic faecal examinations: number (n) and percentage (%) of cats positive for different parasites at macroscopic and microscopic faecal examinations in Site A (Crete island), B (Mykonos island), C (Skopelos island) and D (Athens).

*Cestodes refer to *Joyeuxiella pasqualei* (Sites B-D) */ Diplopylidium nolleri* (Sites C and D) / *Diplopylidium acanthotreta* (Sites B-D).

### Blood

Overall, 148 blood samples were subjected to microscopic and serological assays, i.e. 34, 41, 25, 48 in Sites A-D respectively.

#### Blood smears

Microscopic examination of Giemsa-stained blood smears showed the presence of *Bartonella* spp./*Rickettsia* spp. in four samples, respectively.

#### Serological analyses (Table 2)

Out of these aforementioned four cats (section Blood smears) three were positive at the serology for *Rickettsia* spp. and one were positive for *Bartonella* spp.

**Table 2:** 

Pathogen	Site A (n = 34) n/%	Site B (n = 41) n/%	Site C (n = 25) n/%	Site D (n = 48) n/%	Total (n = 148) n/%
*Bartonella henselae*	22 (64.7)	25 (61)	21 (84)	19 (39.6)	87 (58.8)
*Rickettsia* spp.	18 (53)	14 (34.1)	12 (48)	20 (41.7)	64 (43.2)
*Leishmania infantum*	5 (14.7)	0 (0)	0 (0)	4 (8.3)	9 (6.1)
*Dirofilaria immitis*	3 (8.8)	3 (7.3)	1 (4)	0 (0)	7 (4.7)
*Ehrlichia canis*	0 (0)	0 (0)	0 (0)	3 (6.3)	3 (2)

Serological examinations: number (n) and percentage (%) of cats positive for different arthropod-borne pathogens in Site A (Crete island), B (Mykonos island), C (Skopelos island) and D (Athens).

Overall, the highest percentages of seropositivity were for *B*. *henselae* (58.8%), followed by *Rickettsia* spp. (43.2%), *L*. *infantum* (6.1%), *D*. *immitis* (4.7%) and *E*. *canis* (2%). The seroreactivity against *R*. *typhi* and *R*. *felis* was 41.9% and 6.1% respectively.

#### Genetic examinations (Table 3)

All cats negative by serological analyses were also negative by specific PCR detection (i.e. no false negative results were obtained at the serological tests) for *Rickettsia* spp., *Bartonella* spp., *Leishmania* spp. and Anaplasmataceae. All samples that scored molecularly positive were also positive either at the microscopy or at the serological tests, or both, as detailed below.

**Table 3:** 

Pathogen	Site A (n = 34) n (%) identification	Site B (n = 41) n (%) identification	Site C (n = 25) n (%) identification	Site D (n = 48) n (%) identification	Total (n = 148) n (%) identification
*Rickettsia* spp.	14 (41.2) *R*.*t*.	14 (34.1) *R*.*t*.; *R*.*f*.	11 (44) *R*.*t*.	11 (22.9) *R*.*t*.	50 (33.8) *R*.*t*.; *R*.*f*.
*Bartonella henselae*	1 (2.9) *B*.*h*.	0 (0)	2 (8) *B*.*h*.	4 (8.3) *B*.*h*.; *B*.*c*.	7 (4.7) *B*.*h*.; *B*.*c*.
*Leishmania infantum*	5 (14.7) *L*.*i*.	0 (0)	0 (0)	4 (8.3) *L*.*i*.	9 (6.1) *L*.*i*.
Anaplasmataceae	0 (0)	0 (0)	0 (0)	0 (0)	0 (0)

PCR results: number (n) and percentage (%) of cats positive for different arthropod-borne pathogens in Site A (Crete island), B (Mykonos island), C (Skopelos island) and D (Athens).

Identification of the sequences: *R*.*t*.: *Rickettsia typhi*; *R*.*f*.: *Rickettsia felis*; *L*.*i*.: *Leishmania infantum*; *B*.*h*.: *Bartonella henselae*; *B*.*c*.: *Bartonella clarridgeiae*.

*Rickettsia* spp.: Amplicons of the predicted size of *Rickettsia* spp. were produced for 50 blood samples. Of these samples, 44 were serologically positive to *R*. *typhi*, 2 for *R*. *felis* and 4 to *R*. *typhi*+*R*. *felis*. Fifteen (15) of these amplicons were subjected to the sequencing based on a suitable concentration of amplified DNA on agarose gel, i.e. 10 serologically positive for *R*. *typhi*, 1 for *R*. *felis* and 4 for *R*. *typhi*+*R*. *felis*. The seropositivity of the 10 cats to *R*. *typhi* and of the cat to *R*. *felis* was confirmed at the PCR, with 99% identity with *R*. *typhi* GenBank Accession number U59714.1 and *R*. *felis* GenBank Accession number KT153040.1, respectively. Amplified DNA from 2 cats that seroreacted to *R*. *typhi*+*R*. *felis* showed an identity of 99% with *R*. *typhi* GenBank Accession number U59714.1.

The sequencing of the other 2 amplicons obtained from blood positive for *R*. *typhi*+*R*. *felis* failed.

*Bartonella* spp.: The blood of seven cats, serologically positive for *B*. *henselae*, was also PCR- positive for *Bartonella* spp.; the sequencing confirmed the positivity to *B*. *henselae* in six cats (identity of 100% with GenBank Accession number: KT318618.1) and to *Bartonella clarridgeiae* in one cat (identity of 100% with GenBank Accession number: AB896698.1).

*Leishmania infantum*: The nine cats seropositive for *L*. *infantum* scored also positive upon PCR, with 99–100% identity with *L*. *infantum* GenBank Accession numbers AB896685.1 and HM807524.1.

Anaplasmataceae: The blood of the three cats that seroreacted to *E*. *canis* was PCR-negative.

#### Co-infections and geographic distribution

One hundred and thirty-five cats (90%) were positive for one or more parasites and/or arthropod-borne pathogens upon at least one diagnostic test (Tables 1 and 4). Of the study animals, 44 (29.3%), showed monospecific infections and 91 (60.7%) were positive for more than one pathogen.

**Table 4:** 

	Site A n/% (n = 34)	Site B n/% (n = 43)	Site C n/% (n = 25)	Site D n/% (n = 48)	Total n/% (n = 150)
No. positive (%)	31 (91.2)	39 (90.7)	25 (100)	40 (83.3)	135 (90)
Mono infections (%)	14 (41.2)	14 (32.6)	4 (16)	12 (25)	44 (29.3)
Mixed infections (%)	17 (50)	25 (58.1)	21 (84)	28 (58.3)	91 (60.7)
Coinfections by intestinal parasites*	1 (2.9)	5 (11.6)	1 (4)	4 (8.3)	11 (7.3)
Coinfections by arthropod-borne pathogens	13 (38.2)	6 (14)	6 (24)	8 (16.7)	33 (22)
Coinfections by intestinal parasites* and arthropod-borne pathogens	3 (8.8)	14 (32.6)	14 (56)	16 (33.3)	47 (31.3)

Microscopical fecal and blood examinations: number (n) and percentage (%) of cats positive for monospecific and mixed (i.e. 2–7 different pathogens) infections in Site A (Crete island), B (Mykonos island), C (Skopelos island) and D (Athens). In Site B, 2 cats were examined only for faeces.

VBDs: vector-borne diseases.

*lung parasites are included in the results, as they were detected at the faecal examination.

Site B showed the highest prevalence of infection by zoonotic intestinal parasites (i.e. *T*. *cati*– 32.6% and *D*. *caninum*—7%) (Table 1) while Site A the highest percentage of positivity to vector-borne pathogens of human concern (i.e. *B*. *henselae—*64.7%, *Rickettsia* spp. 53%, *L*. *infantum*—14.7% (Table 2). Site C was the one with the highest prevalence of cats positive for mixed infections including at least one zoonotic pathogen (i.e. 16% *R*. *typhi* + *B*. *henselae*, followed by 17 other different coinfections with 4% of positivity). All 25 cats (100%) examined in site C showed a mono- or a poli-specific infection with at least one zoonotic parasite. The site with the highest number of different combinations of mixed infections including at least one zoonotic pathogen was Site D (n = 21 vs 10, 19 and 18 of Sites A, B and C, respectively).

## Discussion

These results demonstrate that free-roaming and stray cats living in the investigated areas of Greece may be exposed to several parasites and agents transmitted by ectoparasites. These results are likely due to the low levels of treatments with dewormers and ectoparasiticides of cats living in free colonies of the study areas. Various infections here detected are of veterinary and human importance and the vectors transmitting some of them, e.g. fleas, ticks and sand flies, may feed on people.

The common occurrence of co-infections by intestinal helminths might result from a heavy environmental contamination, suggesting that animals (and people in the case of zoonotic parasites) sharing the same habitat are at high risk of infection. Feline geohelminths (i.e. parasites transmitted mainly through contaminated soil), in particular *T*. *cati*, have an important health impact for both animals and humans. The ability of infected cats in contributing to ground and soil contamination with their faeces is relevant because infective roundworm eggs resist for years in the environment [4, 20]. It is very unlikely that the *Toxocara* eggs found in the here examined cat faeces derive from the ingestion of canine faeces by the examined cats. Therefore, it is plausible that all roundworm positive samples were from cats truly infected by *T*. *cati*. Accordingly, the high level of infection by *T*. *cati* (24%) found in the present survey should be taken into a proper account, as they are a potential source of human infection. After *T*. *cati* eggs are passed *via* the animal’s faeces, they mature in few weeks and, if accidentally ingested by people (especially children) migrating larvae may cause *larva migrans* syndromes, e.g. “visceral *larva migrans*” (VLM) and “ocular *larva migrans*” (OLM) [20, 21]. The dog ascarid *Toxocara canis* is largely considered the major agent of these larval syndromes, while the zoonotic role of *T*. *cati* is underestimated [2]. However, there is the indication that *T*. *cati* is associated with both VLM and OLM more frequently than usually thought, especially in causing liver involvement and permanent ocular lesions [22–25].

This study also demonstrates that free-roaming cats from study regions may also acquire mosquito-borne dirofilariosis and flea-transmitted dipylidiosis, and are exposed to different arthropod-borne microorganism, e.g. *Bartonella* spp., *Leishmania* spp. and *Rickettsia* spp..

With regard to arthropod-borne helminths, zoonotic *D*. *immitis* and *D*. *caninum* were here recorded in various cats (Tables 1 and 2). Subcutaneous *Dirofilaria repens* is more frequently involved in causing human diseases in the Old World, but *D*. *immitis*-caused infections are also known, including in Greece [26]. The positivity to *D*. *immitis* in some cats (Table 2) shows that the infection pressure for this parasite may be high under certain circumstances, thus posing a risk for humans. However, the seropositivity of three cats from Crete was not expected, as this area is considered non-endemic for *D*. *immitis* [27]. On the other hand, it should be taken into account that a seropositive result in a cat does not necessarily represent a concurrent infection with adult worms, but it could also indicate an aborted infection, or simply the exposure to infective stages [28]. Hence, these results could simply imply a raising risk of exposure to *D*. *immitis* in non enzootic areas.

Human infections by *D*. *caninum* occur in young people, though rarely [29, 30], after the inadvertent ingestion of infected fleas or their residues. Therefore, the elimination of both this tapeworm and its vectors in cats is of importance to prevent human and animal infections. It should be considered that the percentage of infection by *D*. *caninum* here recorded is likely underestimated, for the low sensitivity of stool examinations in detecting the tapeworm in infected animals [31].

The most important cat VBD in terms of epidemiological and veterinary and human health is bartonellosis caused by *B*. *henselae*. This disease is called cat-scratch fever or cat-scratch disease (CSD), which is transmitted between animals, and from animals to humans, by inoculation of an open wound (e.g. a scratch or a bite), with contaminated flea feces. The infection in people presents with various clinical signs (fever, headache, regional lymphadenopathy, etc.), atypical symptoms and potentially deadly complications in immunocompromised subjects [32, 33]. Cats develop frequently subclinical infections with *B*. *henselae* and often remain undiagnosed and asymptomatic reservoirs [34]. For the aforementioned reasons, free-roaming, stray or feral cats play a crucial role as a source of human infections with *B*. *henselae*. It is thus worthy of note that more than a half of the examined cats (58.8%), coming from all study sites, was *Bartonella*-positive at the serological tests (Table 2). This seropositivity suggests an exposure to the pathogen and does not necessarily indicate that these animals were bacteriemic and infectious for fleas. Nonetheless, the molecular detection of *B*. *henselae* in the blood of some seropositive cats re-inforces their role as a reservoir of bartonellosis. Additionally, one cat was genetically positive to *B*. *clarridgeiae*, which is suspected to be another flea-borne agent of CSD [34–36]. Therefore, an important risk to humans is here shown for these *Bartonella*-positive cats, as carriers of ectoparasites and possible source reservoirs for CSD [37].

Fleas also transmit some rickettsioses of feline and human importance. *Rickettsia felis* is the agent of the flea-borne spotted fever, also known as cat flea typhus. This emerging pathogen is transmitted by the cat flea *Ctenocephalides felis*, although mosquitoes have also been suspected to transmit the bacterium [38]. Cats and other animals (e.g. dogs) may be positive for *R*. *felis*, although animal infections are largely subclinical and the role of mammals as a reservoir needs further confirmatory evidence [39]. In any case, human infections are widely known and are characterized by aspecific symptoms, and neurologic, digestive, and respiratory disorders [40]. Murine typhus is caused by *R*. *typhi*, whose transmission is guaranteed by a classical cycle rat-flea-rat, and a peridomestic cycle involving cats, dogs, other animals and their fleas. Cats and some wild animals are considered reservoirs of infection and *C*. *felis* the principal vector for this pathogen [41]. Although an association between seropositive cats and murine typhus human cases has been suggested [42, 43], no naturally infected cats have been found until recently, when an asymptomatic animal was molecularly diagnosed with *R*. *typhi* in Spain [44]. Human murine typhus is usually a mild febrile illness, though severe and life-threatening sequelae have been described [41, 45]. The high levels of seroreaction to *Rickettsia* spp. (Table 2), along with the molecular confirmation of the *R*. *typhi* and *R*. *felis* DNA upon PCRs (Table 3), show that stray cats of study areas are exposed to flea-borne organisms and are potential carriers of these infections. Importantly, various cats were seropositive for *Rickettsia* spp. and *Bartonella* spp. at the same time, further supporting a high risk for multiple zoonotic flea-borne infections.

*Ehrlichia canis*, i.e. the causative agent of canine monocytic ehrlichiosis, is an important pathogen of dogs worldwide [15]. Conversely, its significance in feline hosts is less clearly defined, although serological and molecular evidence of *E*. *canis*-like infections exists [46, 47]. Tick-borne human ehrlichiosis is usually caused by *Ehrlichia chaffeensis*, which induces a flu-like syndrome, while the role of *E*. *canis* in human infections is yet to be definitively elucidated [48–50]. Although only one cat was here seroreactive for *E*. *canis* (Tables 2 and 3) though negative at the PCR, the risk of exposure to tick-borne pathogens for stray cats in the study areas should be further investigated.

Dogs infected with *L*. *infantum* are a major source of infection for sand fly vectors, which also may bite humans and other animals, e.g. cats. Human leishmaniosis in the Mediterranean Basin may develop in visceral, cutaneous and mucocutaneous diseases that are particularly severe in young, malnourished and immunosuppressed people [51]. Cats are occasional hosts for *L*. *infantum* and it is likely that their role in the epizootiology of leishmaniosis is minor [3]. Positivity to *Leishmania* spp. in felines is rare, though already described in Mediterranean Europe, including Greece [13, 52, 53].

The present detection of cats serologically and molecularly positive for *L*. *infantum* demonstrates that the parasitological pressure with this protozoon in the study areas may represent a potential hazard for animals and people. In fact, *L*. *infantum*-positive cats can be infective for *Phlebotomus perniciosus*, i.e. one of the most important vectors of leishmaniosis [8]. Although the presence of *P*. *perniciosus* is not confirmed in Greece, it cannot be excluded that seropositive cats that are also PCR-positive for *L*. *infantum* (as in the present study) may be capable to transmit the organism to blood-feeding vectors. In the present study, *Leishmania*-positive cats were from Sites A (14.7%) and D (8.3%) where sand flies are present and the parasite is well established [54–55]. Importantly, Sites A and D are in areas endemic for visceral leishmaniosis [55], thus further studies aiming at understanding the role of cats in the epizootiology of the parasite should be encouraged.

Overall, information regarding exposure risk to zoonoses and prevalence of pathogens among various cat populations is still insufficient in vast geographic areas, especially for VBDs [3]. Therefore, the present work adds novel information on the occurrence of zoonotic parasites and arthropod-borne pathogens of cats living in areas of Greece. The independent lifestyle of cats, especially in free-roaming colonies, may result in a high environmental contamination with parasite elements, e.g. roundworm eggs [4], and in an increased risk for humans of contact with cat ectoparasites or direct disease transmission, e.g. bartonellosis [31]. Most of the VBDs here recorded are often clinically silent and undetected in cats, yet they can cause morbidity and even mortality in humans. The presence of antibodies against important VBDs and/or of circulating microorganism DNA demonstrates that cats are frequently exposed to arthropod vectors and transmitted diseases in the geographic regions under study.

Given that cats infected by zoonotic parasites and/or VBDs may appear healthy, it is likely that population-level exposure to these diseases is often overlooked without a focused and constant surveillance.

Routine surveillance is required in touristic areas because movements of pets pose a substantial risk in the spreading of pathogens because i) they may be introduced from enzootic to free regions when infected pets travel with their owners, ii) pets travelling to enzootic areas may acquire pathogens from visited environments or from local animals, and introduce them in free areas when they go home. Importantly, many zoonotic pathogens (e.g. *Bartonella* spp., *Rickettsia* spp.) infecting cats and various ectoparasites (e.g. ticks and fleas) are also capable to infect/infest dogs. This is of importance if one considers that dogs frequently travel with their families during holidays. Therefore, control measures are fundamental towards the prevention and control of zoonotic infections and external parasitoses in both dogs and cats, *via* planned treatments and preventative measures with broad-spectrum and efficacious parasiticide formulations. At the same time, it is crucial to educate the general public, veterinary practitioners, and public health officials on the factual risks for diseases potentially associated with free-roaming cats, in order to encourage safe movement of people and pets and to do not unreasonably discourage direct contacts with local cats.
